# Genomic selection models for directional dominance: an example for litter size in pigs

**DOI:** 10.1186/s12711-018-0374-1

**Published:** 2018-01-26

**Authors:** Luis Varona, Andrés Legarra, William Herring, Zulma G. Vitezica

**Affiliations:** 1 Departamento de Anatomía, Embriología y Genética Animal, Universidad de Zaragoza, 50013 Saragossa, Spain; 2 Instituto Agroalimentario de Aragón (IA2), 50013 Saragossa, Spain; 3 INRA, GenPhySE (Génétique, Physiologie et Systèmes d’Elevage), 31326 Castanet-Tolosan, France; 4 PIC North America, 100 Bluegrass Commons Blvd., Suite 2200, Hendersonville, TN 37075 USA; 5 INP, ENSAT, GenPhySE (Génétique, Physiologie et Systèmes d’Elevage), Université de Toulouse, 31326 Castanet-Tolosan, France

## Abstract

**Background:**

The quantitative genetics theory argues that inbreeding depression and heterosis are founded on the existence of directional dominance. However, most procedures for genomic selection that have included dominance effects assumed prior symmetrical distributions. To address this, two alternatives can be considered: (1) assume the mean of dominance effects different from zero, and (2) use skewed distributions for the regularization of dominance effects. The aim of this study was to compare these approaches using two pig datasets and to confirm the presence of directional dominance.

**Results:**

Four alternative models were implemented in two datasets of pig litter size that consisted of 13,449 and 11,581 records from 3631 and 2612 sows genotyped with the Illumina PorcineSNP60 BeadChip. The models evaluated included (1) a model that does not consider directional dominance (Model SN), (2) a model with a covariate *b* for the average individual homozygosity (Model SC), (3) a model with a parameter *λ* that reflects asymmetry in the context of skewed Gaussian distributions (Model AN), and (4) a model that includes both *b* and *λ* (Model Full). The results of the analysis showed that posterior probabilities of a negative *b* or a positive *λ* under Models SC and AN were higher than 0.99, which indicate positive directional dominance. This was confirmed with the predictions of inbreeding depression under Models Full, SC and AN, that were higher than in the SN Model. In spite of differences in posterior estimates of variance components between models, comparison of models based on LogCPO and DIC indicated that Model SC provided the best fit for the two datasets analyzed.

**Conclusions:**

Our results confirmed the presence of positive directional dominance for pig litter size and suggested that it should be taken into account when dominance effects are included in genomic evaluation procedures. The consequences of ignoring directional dominance may affect predictions of breeding values and can lead to biased prediction of inbreeding depression and performance of potential mates. A model that assumes Gaussian dominance effects that are centered on a non-zero mean is recommended, at least for datasets with similar features to those analyzed here.

**Electronic supplementary material:**

The online version of this article (10.1186/s12711-018-0374-1) contains supplementary material, which is available to authorized users.

## Background

Since the availability of dense genotyping panels [1], genomic prediction [2, 3] has become a very successful strategy for the prediction of breeding values of candidates for selection. Genomic prediction methods are based on the evaluation of the additive substitution effects of markers that capture a large part of the dominance and higher-order interaction effects [4]. However, estimating dominance effects may be relevant because their estimates can be used to allocate mates among candidates for selection [5, 6].

Two approaches have been suggested to estimate the effects of dominance in genomic prediction methods. The first [7] directly models the additive (a) and dominance (d) effects, while for the second, Vitezica et al. [8] proposed to include allele substitution (α) and dominance deviation (δ) effects in order to compute appropriate breeding values. However, both these approaches impose a Gaussian regularization of additive and dominance effects that forces a symmetric distribution of the posterior estimates.

Nevertheless, the classical theory of quantitative genetics [9] argues that inbreeding depression and heterosis are based on the presence of directional dominance (i.e., a higher percentage of positive than negative dominance effects) and this contrasts with the assumption of symmetry of the above-described procedures. This discrepancy can be overcome in at least two ways: (1) by assuming that the mean of dominance effects differ from zero, which leads to the inclusion of a covariate for the average individual homozygosity in the statistical model, and (2) by using skewed distributions for the regularization of dominance effects. The first approach can be called regression on genomic inbreeding and it was empirically used by Sun et al. [6], Silió et al. [10], Aliloo et al. [11] and Zeng et al. [12], and proved by Xiang et al. [13]. As far as we are aware, the second approach was never applied in the field of animal genetics, although it may be of considerable value since it ensures that the most frequent dominance effects are close to zero. In contrast, regression on genomic inbreeding implies that the mean and mode of the dominance effects are equal, although they may differ from zero. In the statistical literature, there is a broad corpus on the specification of skewed distributions [14, 15] and, among them, the family of skew-elliptical distributions defined by Sahu et al. [16] can be easily implemented in Bayesian regression using Markov chain Monte Carlo (MCMC) techniques [17].

The objectives of this study were: (1) to develop a genomic best linear unbiased prediction (BLUP) model that uses a prior skewed distribution for dominance effects; (2) to compare it with the model with inclusion of a covariate for inbreeding proposed by Xiang et al. [13]; and (3) to confirm the presence of directional dominance for pig litter size.

## Methods

### Data

The data used in this study were from two unrelated pig lines provided by Genus plc (Hendersonville, TN, USA). Genotypes for all sows were generated using the Illumina PorcineSNP60 BeadChip (Illumina, San Diego). After quality control, i.e. excluding genotypes from single nucleotide polymorphisms (SNPs) with a minor allele frequency lower than 0.05 and a call rate lower than 0.95 in each population, 37,900 and 37,011 genotypes for SNPs remained for lines 1 and 2, respectively. Individuals with a call rate lower than 0.95 were also removed. Finally, the number of sows included in the analysis were 3631 and 2612 for lines 1 and 2, respectively. In total, 13,449 and 11,581 records on litter size (number of piglets born alive) were available for these sows, with an average litter size of 11.7 ± 2.9 and 12.4 ± 3.0 for lines 1 and 2, respectively.

### Genomic prediction models

The first step was the definition of a Full Model that included both approaches for directional dominance, i.e. regression on genomic inbreeding and a skewed distribution for SNP effects). Then, a subset of model parameters was set to 0 in order to identify reduced models. The Full Model was:

$${\mathbf{y}} = {\mathbf{1}}\mu + {\mathbf{h}}b + {\mathbf{Xt}} + {\mathbf{Wr}} + {\mathbf{Qc}} + {\mathbf{Za}} + {\mathbf{Kd}} + {\mathbf{e}},$$

where ${\mathbf{y}}$ is the vector of phenotypic records, $\mu$ is the general mean, $b$ is a covariate that can be interpreted as inbreeding depression or heterosis, ${\mathbf{t}}$ is a vector of order of parity effects (4 levels − 1st, 2nd, 3rd and > 3rd), ${\mathbf{r}}$ is a vector of farm-year-month of farrowing effects (3163 levels for line 1 and 4293 for line 2), ${\mathbf{c}}$ is a vector of permanent environmental effects (3631 and 2612 levels for lines 1 and 2, respectively), ${\mathbf{a}}$ and ${\mathbf{d}}$ are vectors of additive and dominance effects (37,900 and 37,011 levels), and ${\mathbf{e}}$ is a vector of residuals. Furthermore, ${\mathbf{h}}$ is a vector of the average SNP homozygosity of the individuals and ${\mathbf{X}}$, ${\mathbf{W}}$, ${\mathbf{Q}}$, ${\mathbf{Z}}$ and ${\mathbf{K}}$ are incidence matrices that link the phenotypic records with ${\mathbf{t}}$, ${\mathbf{r}}$, ${\mathbf{c}}$, ${\mathbf{a}}$ and ${\mathbf{d}}$, respectively. Under the Bayesian paradigm, prior distributions were uniform for $\mu$, $b,$ and for each element of ${\mathbf{t}}$, univariate Gaussian for each element of ${\mathbf{r}}$, ${\mathbf{c}}$ and ${\mathbf{a}}$, and skew Gaussian for each element of ${\mathbf{d}}$. Finally, prior distributions for the variances of farm-year-month of farrowing ($\sigma_{r}^{2}$), permanent environmental ($\sigma_{c}^{2}$), additive ($\sigma_{a}^{2}$), dominance ($\sigma_{d}^{2}$), and residual effects ($\sigma_{e}^{2}$) were scaled inverted Chi square (see “Appendix” for a full description of the Bayesian inference). It should be noted that directional dominance comprises two model parameters, one covariate for the average SNP homozygosity ($b$) and one asymmetry parameter ($\lambda$) that is involved in the skew Gaussian prior distribution of dominance effects.

Based on the Full Model, three reduced models were defined as follows:

- Model SC: symmetric dominance effect with the inbreeding depression covariate, i.e. the asymmetry parameter ($\lambda$) was set to zero. This model was equivalent to that defined by Xiang et al. [13].
- Model AN: asymmetric dominance effect without the inbreeding depression covariate, i.e. covariate $b$ was set to zero.
- Model SN: symmetric dominance effect without the inbreeding depression covariate, i.e. both asymmetry parameter $\lambda$ and covariate $b$ were set to zero. This model was equivalent to that defined by Su et al. [5].

The four models (Full, SC, AN, SN) were analyzed using a Gibbs sampler [18] (see “Appendix” for a full description) that provided posterior distributions for all unknowns in the model, i.e. individual breeding values ($s_{a}$) and dominance deviations ($s_{d}$), additive and dominance variances (${\text{V}}_{\text{A}}$ and ${\text{V}}_{\text{D}}$), and the expected inbreeding depression per percentage of inbreeding (${\text{I}}_{\text{D}}$). Models SC, AN and SN were analyzed by five chains of 75,000 iterations, after discarding the first 25,000. Each chain used a different random seed. As the convergence of the Full Model was clearly the worst, the Gibbs sampler implementation for this model was set to five chains of 250,000 iterations, after a burn-in of the first 50,000. Convergence and effective sample size were checked using the standard procedures [19] with CODA package [20] and by visual inspection of the chains. Finally, models were compared using the deviance information criteria (DIC) [21] and the logarithm of the conditional predictive ordinate (LogCPO) [22] (see “Appendix” for a full description).

## Results

The results of the convergence and the effective size of the MCMC chains are presented in Additional file 1: Tables S1 and S2. The average number of iterations required until convergence was computed using the Raftery and Lewis approach [23] and ranged from 93.0 (for parameter $b$ in the SC Model for line 1) to 9204.0 ($b$ in the Full Model for line 1). The estimated effective sample size (EFS) of the MCMC chains [24] ranged from 82 (${\text{d}}^{2}$ in the SN Model for line 2) to 16,510 (${\text{h}}^{2}$ in the Full Model for line 1). Finally, the required numbers of samples to achieve an accuracy of 0.1 for the 0.5 quantile with a probability of 0.95 were calculated using the Raftery and Lewis approach [23] and ranged from 3210 ($b$ in the SC Model for line 1) to 290,280 ($b$ in the Full Model for line 1).

Posterior mean estimates (and posterior deviations) of variance components, asymmetry parameters, and expected inbreeding depression and results of the comparison of models are in Tables 1 and 2 for lines 1 and 2, respectively. Posterior estimates of the variance of the additive effects ($\sigma_{a}^{2}$) under Model SN were equal to 0.394 × 10^−4^ and 0.678 × 10^−4^ for lines 1 and 2, respectively. Compared with the SN Model, these estimates were slightly lower for the AN Model (0.345 × 10^−4^ and 0.617 × 10^−4^), those for the SC Model were moderately higher (0.439 × 10^−4^ and 0.701 × 10^−4^) (Tables 1 and 2) and those for the Full Model were similar (0.381 × 10^−4^ and 0.615 × 10^−4^).

**Table 1:** Posterior mean (and posterior standard deviation) estimates for variance components, asymmetry parameters, inbreeding depression, ratios of additive and dominance variation and criteria for model comparison for line 1

	Model			
	SN	AN	SC	Full
$b$	–	–	− 12.153 (1.746)	− 7.950 (7.527)
$\sigma_{a}^{2}$ (× 10^−4^)	0.394 (0.062)	0.345 (0.061)	0.439 (0.065)	0.381 (0.067)
$\sigma_{d}^{2}$ (× 10^−4^)	0.369 (0.101)	0.769 (0.108)	0.122 (0.095)	0.536 (0.236)
$\sigma_{r}^{2}$	0.160 (0.043)	0.161 (0.043)	0.160 (0.043)	0.161 (0.043)
$\sigma_{c}^{2}$	0.478 (0.089)	0.308 (0.084)	0.572 (0.089)	0.394 (0.116)
$\lambda_{d}$ (× 10^−3^)	–	0.380 (0.078)	–	0.135 (0.244)
$\sigma_{e}^{2}$	6.569 (0.099)	6.570 (0.099)	6.567 (0.099)	6.568 (0.098)
${\text{I}}_{\text{D}}$	− 0.016 (0.005)	− 0.044 (0.006)	− 0.045 (0.006)	− 0.045 (0.006)
${\text{V}}_{\text{A}}$	0.679 (0.101)	0.862 (0.1174)	0.832 (0.110)	0.859 (0.158)
${\text{V}}_{\text{D}}$	0.597 (0.165)	1.326 (0.211)	0.415 (0.165)	1.013 (0.343)
${\text{h}}^{2}$	0.080 (0.011)	0.093 (0.015)	0.097 (0.011)	0.095 (0.015)
${\text{d}}^{2}$	0.070 (0.018)	0.143 (0.019)	0.048 (0.018)	0.111 (0.034)
LogCPO	− 32,508.88	− 32,513.61	− 32,498.72	− 32,517.83
DIC	64,939.52	64,948.21	64,920.97	64,947.08

$b$ is the covariate with individual homozygosity, $\sigma_{a}^{2}$ and $\sigma_{d}^{2}$ are the variance of the additive and dominance SNP effects, $\sigma_{r}^{2}$ is the variance of the permanent environmental effects, $\sigma_{c}^{2}$ is the variance of the farm-year-month effects, $\lambda_{d}$ is the asymmetry parameters for the dominance effects, $\sigma_{e}^{2}$ is the residual variance, ${\text{I}}_{\text{D}}$ is the inbreeding depression per percentage of inbreeding, ${\text{V}}_{\text{A}}$ and ${\text{V}}_{\text{D}}$ are the additive and dominance variance, ${\text{h}}^{2}$ and ${\text{d}}^{2}$ are the heritability and the ratio of dominance variance, LogCPO is the logarithm of the conditional predictive ordinate and DIC is the deviance information criterion

**Table 2:** Posterior mean (and posterior standard deviation) estimates for variance components, asymmetry parameters, inbreeding depression, ratios of additive and dominance variation and criteria for model comparison for line 2

	Model			
	SN	AN	SC	Full
$b$	–	–	− 6.479 (2.289)	1.726 (5.845)
$\sigma_{a}^{2}$ (× 10^−4^)	0.678 (0.091)	0.617 (0.092)	0.701 (0.095)	0.615 (0.096)
$\sigma_{d}^{2}$ (× 10^−4^)	0.430 (0.170)	0.872 (0.169)	0.334 (0.154)	0.993 (0.322)
$\sigma_{r}^{2}$	0.299 (0.060)	0.296 (0.062)	0.299 (0.061)	0.297 (0.060)
$\sigma_{c}^{2}$	0.580 (0.123)	0.380 (0.114)	0.614 (0.118)	0.333 (0.148)
$\lambda_{d}$ (× 10^−3^)	–	0.249 (0.096)	–	0.307 (0.209)
$\sigma_{e}^{2}$	6.630 (0.109)	6.635 (0.110)	6.631 (0.109)	6.635 (0.108)
${\text{I}}_{\text{D}}$	− 0.008 (0.005)	− 0.028 (0.008)	− 0.025 (0.008)	− 0.029 (0.008)
${\text{V}}_{\text{A}}$	1.100 (0.125)	1.170 (0.171)	1.152 (0.135)	1.198 (0.174)
${\text{V}}_{\text{D}}$	0.669 (0.263)	1.377 (0.274)	0.574 (0.241)	1.537 (0.464)
${\text{h}}^{2}$	0.119 (0.013)	0.118 (0.015)	0.124 (0.013)	0.120 (0.015)
${\text{d}}^{2}$	0.072 (0.027)	0.139 (0.024)	0.061 (0.024)	0.152 (0.040)
LogCPO	− 28,176.11	− 28,176.62	− 28,174. 84	− 28,180.68
DIC	56,250.06	56,251.39	56,247.95	56,258.66

$b$ is the covariate with individual homozygosity, $\sigma_{a}^{2}$ and $\sigma_{d}^{2}$ are the variance of the additive and dominance SNP effects, $\sigma_{r}^{2}$ is the variance of the permanent environmental effects, $\sigma_{c}^{2}$ is the variance of the farm-year-month effects, $\lambda_{d}$ is the asymmetry parameters for the dominance effects, $\sigma_{e}^{2}$ is the residual variance, ${\text{I}}_{\text{D}}$ is the inbreeding depression per percentage of inbreeding, ${\text{V}}_{\text{A}}$ and ${\text{V}}_{\text{D}}$ are the additive and dominance variance, ${\text{h}}^{2}$ and ${\text{d}}^{2}$ are the heritability and the ratio of dominance variance, LogCPO is the logarithm of the conditional predictive ordinate and DIC is the deviance information criterion

A different pattern was observed for the variance of dominance effects ($\sigma_{d}^{2}$), with posterior mean estimates being equal to 0.369 × 10^−4^ and 0.430 × 10^−4^ for lines 1 and 2, respectively, for Model SN (Tables 1 and 2). Models that allowed for asymmetry of dominance effects (AN and Full) provided higher posterior mean estimates of the variance of dominance effects (0.769 × 10^−4^ and 0.536 × 10^−4^ for line 1 and 0.872 × 10^−4^ and 0.993 × 10^−4^ for line 2, respectively) than the SC Model (0.122 × 10^−4^ and 0.334 × 10^−4^ for lines 1 and 2, respectively).

Because of the above results, estimates of additive genetic variance (${\text{V}}_{\text{A}}$) and narrow sense heritability (${\text{h}}^{2}$) were higher for Models AN and Full than for Models SN and SC (Tables 1 and 2). In contrast, posterior mean estimates of the variance of dominance deviations (${\text{V}}_{\text{D}}$) and percentage of dominance variation (${\text{d}}^{2}$) were lower for Models SN and SC than for Models AN and Full (Tables 1 and 2).

Estimates of the variance of farm-year month effects ($\sigma_{r}^{2}$) and of residuals ($\sigma_{e}^{2}$) were consistent between models, ranging from 0.160 to 0.161 for line 1 and from 0.296 to 0.299 for line 2 for the farm-year-month variance and from 6.567 to 6.570 and from 6.630 to 6.635 for the residual variance for lines 1 and 2, respectively. However, the estimates of the variance of permanent environmental effects ($\sigma_{c}^{2}$) differed substantially between models (Tables 1 and 2), with posterior mean estimates for the SN and SC Models being the highest (0.478 and 0.572 for line 1 and 0.580 and 0.614 for line 2, respectively) and decreasing when asymmetry was allowed, reaching the lowest estimates for Models AN (0.308 and 0.380 for lines 1 and 2, respectively) and Full (0.394 and 0.333).

**Fig. 1:**
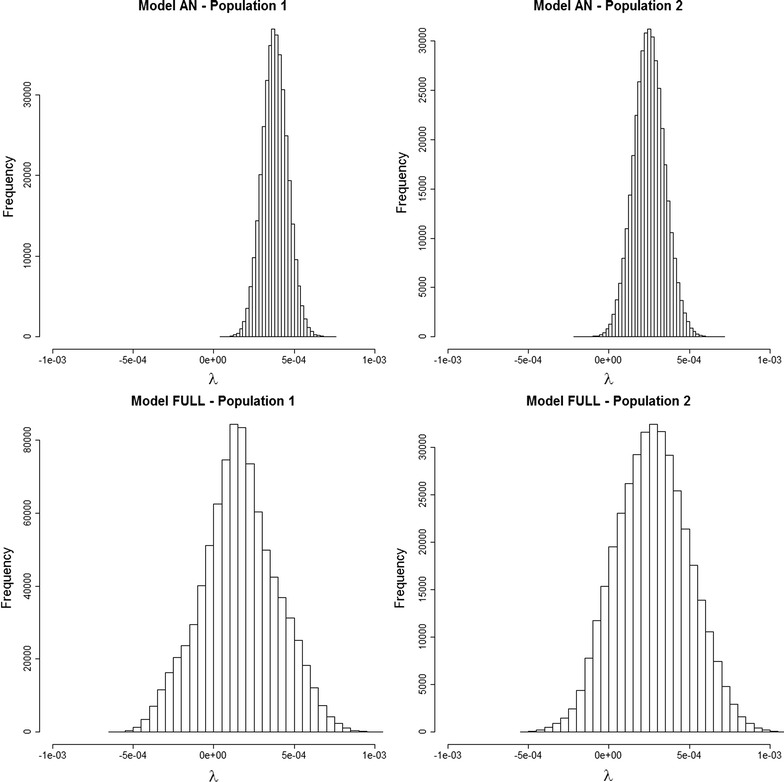
Posterior distribution of the asymmetry parameter (λ) under Models AN and Full for lines 1 and 2

Posterior mean estimates of the asymmetry parameter for dominance effects ($\lambda$) were all positive (Tables 1 and 2 and Fig. 1) and ranged from 0.135 (line 1 and Model Full) to 0.380 (line 1 and Model AN). However, it should also be noted that posterior probabilities of a positive value for $\lambda$ were higher than 0.999 for Model AN, while the highest posterior density regions at 95% (HPD95) for $\lambda$ included zero for the Full Model for both lines.

**Fig. 2:**
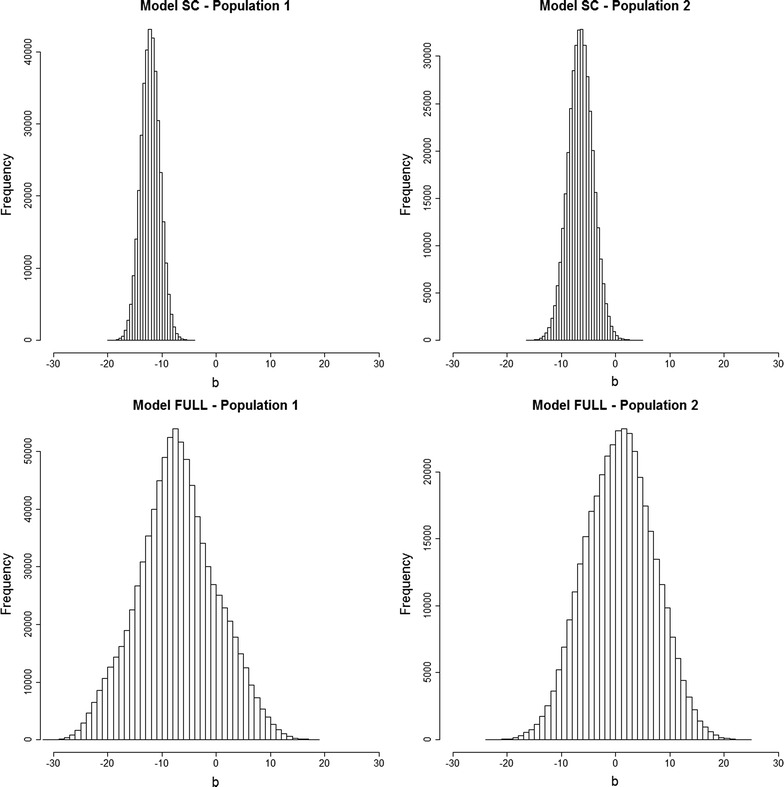
Posterior distribution of the covariate for individual homozygosity (b) under Models SC and Full for lines 1 and 2

The regression coefficient on individual homozygosity ($b$) was estimated with Models SC and Full (Tables 1 and 2 and Fig. 2). With the SC Model, posterior mean estimates of $b$ were clearly negative (− 12.15 and − 7.95 for lines 1 and 2, respectively), but equal to − 5.72 and 1.73 for lines 1 and 2 for the Full Model. It should also be noted that posterior standard deviations were higher for the Full than for the SC Model. The HPD95 regions for $b$ included zero for the Full Model, but posterior probabilities of negative values were always higher than 0.99 for Model SC.

**Fig. 3:**
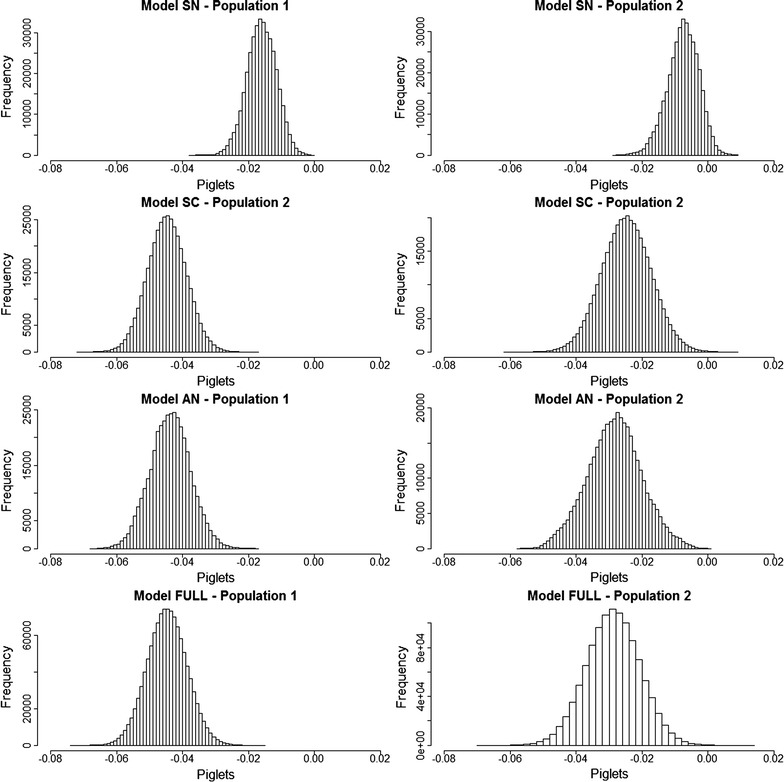
Posterior distribution of the expected inbreeding depression for an inbreeding level of 0.10 for lines 1 and 2

Results for the expected inbreeding depression (${\text{I}}_{\text{D}}$) per percentage of inbreeding are in Tables 1 and 2 and Fig. 3. Posterior mean (and posterior standard deviation) estimates of ${\text{I}}_{\text{D}}$ for the SN Model were − 0.016 (0.005) piglets for line 1 and − 0.008 (0.005) piglets for line 2. However, posterior mean (and posterior standard deviation) estimates for remaining Models (AN, SC and Full) were remarkably lower, being − 0.044 (0.006), − 0.045 (0.006), and − 0.045 (0.006) for line 1 and − 0.028 (0.008), − 0.025 (0.008) and − 0.029 (0.008) for line 2.

Correlations of estimates for the SNP additive ($a$) and dominance ($d$) effects and for breeding values ($s_{a}$) and dominance deviations ($s_{d}$) between the four models of analysis are in Additional file 2: Tables S3 and S4. Correlations of estimates of the additive and dominance effects between models were always higher than 0.990 and correlations of estimates of breeding values and dominance deviations between models were also close to 1. However, it should be noted that the correlations between the estimated breeding values from the SN Model and the dominance deviations from the AN Model with the estimates from the remaining models were remarkably lower than those from the other models. In the first case, they ranged from 0.933 to 0.944 in line 1 and from 0.794 to 0.842 in line 2 and in the second case, from 0.769 to 0.944 in line 1 and from 0.702 to 0.857 in line 2.

Results of the model comparison tests (logCPO and DIC) are also in Tables 1 and 2. In both lines, the model with the best fit for both tests was the SC Model, followed by the SN and AN Models. The Full Model had the worst fit.

## Discussion

The advent of dense genotyping information has allowed the development of models for genomic evaluation [3] that have revolutionized the field of animal breeding during the last decade. Most models for genomic evaluation are designed to deal with the classical statistical problem of large $p$ and small $n$, because the number of parameters to evaluate is frequently larger than the number of phenotypic data. The most common approach for dealing with this problem is the use of some kind of regularization of the effects of SNPs [25]. Several approaches have been suggested, ranging from simple Gaussian regularization [2] to more complex models that involve t-shaped [2], double exponential [26, 27], or mixtures of distributions [2, 28, 29]. However, all these methods of regularization use symmetric distributions that, from a Bayesian perspective, imply that marker effects are centered at zero. This assumption seems reasonable for the additive or substitution effects, but it is not so clear for dominance effects. In fact, the classical theory of quantitative genetics attributes the phenomenon of inbreeding depression (or heterosis) to the presence of directional dominance or, in other words, a positive average of dominance effects, jointly with a decrease (or increase) in the degree of heterozygosity [9]. In this study, we considered two approaches to model directional dominance in genomic evaluation methods. The first assumed a prior distribution for dominance effects that allowed a mean that was different from 0, i.e. Model SC, following the work of Xiang et al. [13]; the second assumed that dominance effects followed a skew Gaussian distribution that has a higher probability of positive (or negative) effects, i.e. Model AN. Finally, both approaches were combined into a Full Model.

All models were implemented using a Gibbs sampler. The analysis of the MCMC chains indicated that convergence was achieved with the proposed burn-in for all models and both lines (25,000 iterations for AN, SC and SN Models and 50,000 for the Full Model). Nevertheless, the EFS was heterogeneous across parameters and models. In general, the EFS of the variance of dominance effects was smaller than that of the variance of additive effects, and the EFS of the parameters related with directional dominance ($b$ and $\lambda$) were very large for the AN and SN Models and remarkably smaller for the Full Model. Nevertheless, the sizes of the five Gibbs sampler chains (5 × 75,000 iterations for AN, SN and SC Models and 5 × 200,000 for the Full Model) were always larger than the length required for estimation of the 0.5 quantile of the posterior distributions with an accuracy of 0.1 and with a probability of 0.95, based on the Raftery and Lewis approach [23].

### Evidence of directional dominance

Results from Models SC and AN provided clear evidence of directional dominance for both lines (Figs. 1 and 2); posterior distributions of the regression coefficient on individual homozygosity ($b$ in Model SC) and the asymmetry parameter ($\lambda$ in Model AN) did not include zero in the highest posterior density at 99%. These results confirm the presence of directional dominance for litter size in pigs and they are in line with extensive reports on positive estimates for inbreeding depression and heterosis in the literature [30, 31]. However, results from the Full Model were not so clear because it suffered from some degree of statistical confounding of $b$ and $\lambda$, as observed in the strong posterior correlation (0.91) between the Gibbs samples of $b$ and $\lambda$ (see Additional file 3: Figures S1 and S2). As a consequence, their posterior distributions were wider and they included zero in the HPD at 95% for $b$ and $\lambda$ (Figs. 1 and 2) and convergence and EFS for both these parameters were worse than with the SC and AN Models (see Additional file 1: Tables S1 and S2).

Models that allow the presence of directional dominance (SC, AN, Full) were able to predict the expected inbreeding depression (${\text{I}}_{\text{D}}$) in populations that had a low range of levels of genealogical inbreeding. This approximation uses the classical additive model of inbreeding depression [9] but replacing dominance effects of causal polymorphisms with dominance effects of SNPs. In this approach, a linear relationship between inbreeding and inbreeding depression is assumed. Results were presented as the expected inbreeding depression per percentage of inbreeding. In the analyzed populations, the expected inbreeding depression coefficients under these models were around − 0.045 and between − 0.025 and − 0.028 piglets in lines 1 and 2, respectively. These results concur with those of Vitezica et al. [32], who also reported larger estimates of inbreeding depression in line 1 than in line 2 and they are close to the estimates of inbreeding depression for litter size in other pig populations [33–35]. In contrast, the estimates provided by the SN Model were substantially closer to 0, i.e. − 0.016 and − 0.008 piglets for lines 1 and 2, respectively. This may indicate that models that do not allow for directional dominance, such as the SN Model, cannot predict the magnitude of inbreeding depression (or heterosis) correctly and, thus, lead to biases if they are used for the prediction of future mate performance and mate allocation [5].

Nevertheless, there were some remarkable differences between the results obtained for the two lines, which are interesting to analyze further. Evidence of directional dominance was larger for line 1 than for line 2 for both approaches (estimates of − 12.15 vs. − 6.48 for $b$ in Model SC and of 0.38 vs. 0.25 for $\lambda$ in Model AN), although posterior estimates of the dominance variance were lower for line 1 for all models. This suggests that the magnitude of directional dominance (or inbreeding depression) is not necessarily related to the amount of dominance variance estimated from resemblance between relatives. In fact, in the presence of inbreeding, the total genetic variance is split into five components [36, 37]: the additive and dominance genetic variances in the base population, the dominance genetic variance between homozygous individuals, the covariance between additive and dominance effects between homozygous individuals, and the square of the inbreeding depression. Traditional approaches to estimate dominance variance using genealogical [38, 39] or genomic dominance relationships [32] only take the additive and dominance variance in the base population into account and ignore the remaining variance components. It is possible that the presence of directional dominance also allows some of the other variance components that are not considered under the assumption of multivariate normality to be captured.

Of particular significance is the fact that the estimates of the variance of dominance effects differed substantially between models. Lower estimates were obtained with the SC Model, whereas estimates from Models SN, AN and Full were higher. The cause of the inflation of dominance effects under the last three models may be the restrictions imposed by the assumed prior distributions. Under Model SN, the prior distribution forced the mean and mode of effects to be centered at zero. Thus, if directional dominance exists, specific estimates of the effects of SNPs would attempt to accommodate this, which would lead to an increase in the variance of dominance effects. Model AN allowed the presence of more positive (or negative) dominance effects but it forced the mode of the distributions to be close to zero. Estimates of the effects of SNPs for Model AN may be even larger than for Model SN, but as the prior distribution forced them to have a mode close to zero, the variance of dominance effects was also inflated in Model AN. Furthermore, the increase in the variance of dominance effects in Models SN, AN and Full with respect to Model SC was compensated by a corresponding decrease in the permanent environmental variance, as pointed out in other studies [40, 41]. Thus, the estimate of the permanent environmental variance was the largest for Model SC for both lines.

The differences between models were also reflected in the correlations of estimates of breeding values and dominance deviations between models. Although the correlations for estimates of SNP additive and dominance effects between models were very high (see Additional file 2: Tables S3 and S4), the correlations for estimates of breeding values and dominance deviations provided some exceptions. For breeding values, estimates from the model that did not consider directional dominance (Model SN) had lower correlations with estimates from the other models (SC, AN and Full). This suggests that the inclusion of directional dominance with either of the two approaches would result in substantial changes in the ranking of individuals based on estimates of breeding values, which may have consequences for breeding decisions. In addition, the correlations between estimates of dominance deviations from Model AN with those from the other models were also lower (0.70–0.94), which may imply that the use of skewed prior distributions affects estimation of dominance deviations and the prediction of performance of future individuals (or crosses).

### Comparison of models

The best model based on the two criteria used for comparison of models was the SC Model, followed by the SN and AN Models; the Full Model provided the worst fit for both lines. Model SN does not consider directional dominance and thus, it was penalized relative to Model SC. Models AN and Full were equally able to capture directional dominance since they led to similar estimates of inbreeding depression. However, they were penalized because the number of unknowns in these models is larger than in Model SC; they estimate $\lambda$ and one auxiliary variable for the dominance effect of each SNP.

In the light of these results, the main finding of our study is that Model SC, as defined by Xiang et al. [13], is recommended for the analysis of traits when directional dominance (or inbreeding depression) is expected and when resulting estimates of dominance effects are used for prediction of performance of future mates and mate allocation [5]. This recommendation is strengthened by the ease with which the SC Model can be formulated based on the genomic dominance relationship matrix [8], which helps to reduce the computational burden and directly provides predictions of additive and dominance effects for each individual.

However, the application of skewed distributions should not be completely discarded for new lines of research. First, we assumed that the additive and dominance effects were independent, although it is possible to use multivariate asymmetric distributions [16], as in the models of Wellman and Bennewitz [42], which consider a relationship between the magnitudes of additive and dominance effects. Second, the assumption of Gaussian distributions can be replaced by the asymmetric version of any other distribution, such as t-shape or double exponential distribution, leading to asymmetric versions of the Bayes B [3] or Bayesian Lasso [26] approaches. These approaches may avoid the large increase in the variance of dominance effects since most of the estimates of the dominance effects of SNPs will be forced to be zero [3] or closer to zero [26] than with a prior Gaussian distribution.

Finally, all the approaches described here assume that directional dominance is homogeneous along the genome. However, there is evidence in the literature of local differences in the causes of inbreeding depression across the genome [43, 44]. Further research is needed to investigate this phenomenon and, also, to model additional causes of inbreeding depression (or heterosis), such as epistatic interactions [45].

## Conclusions

The results of our study confirm the presence of positive directional dominance for litter size in two lines of pigs. Ignoring this in genomic evaluation models with dominance effects alters the prediction of breeding values and may cause bias in the prediction of inbreeding depression (or heterosis) and of the performance of future mates. These effects can be avoided by using two alternative models, one that includes a non-zero mean of dominance effects and another that uses skewed prior distributions for them, with the latter providing a better fit. Thus, this approach should be recommended for modeling dominance effects, at least for datasets that have similar features as those analyzed here.

### Additional files

**Additional file 1: Tables S1 and S2.** Convergence and effective sample size of the Gibbs sampler. These tables include the results of the required burn-in and required sample size and the estimates of the effective sample size of the Gibbs sampler for Models SN, SC, AN and Full in lines 1 (Table S1) and 2 (Table S2).

**Additional file 2: Tables S3 and S4.** Correlations between estimates from Models SN, AC, AN and Full. These tables present the correlations between estimates of SNP additive (*a*) and dominance (*b*) effects, individual breeding values (*c*) and dominance deviations (*d*) with Models SN, SC, AN and Full in lines 1 (Table S3) and 2 (Table S4).

**Additional file 3: Figures S1 and S2.** Plots of the Gibbs sampler chains for the Full Model. These figures include the plots of the five Gibbs sampler chains for the covariate with individual heterozygosity (*b*) and the asymmetry parameter (*λ*) and bivariate plots of the Gibbs samples for the Full Model in lines 1 (Figure S1) and 2 (Figure S2).

## Appendix

### The model

First, we define the joint posterior distribution of all the unknowns in the model as:

$$\begin{aligned} & p\left( {\mu , b, {\mathbf{t}}, {\mathbf{r}}, {\mathbf{c}},\varvec{ }{\mathbf{a}}, {\mathbf{d}}, \sigma_{r}^{2} , \sigma_{c}^{2} , \sigma_{a}^{2} , \sigma_{d}^{2} , \lambda , \left. {\sigma_{e}^{2} } \right|{\mathbf{y}}} \right)\infty \\ & \left( {\left. {\mathbf{y}} \right|\mu , b, {\mathbf{t}}, {\mathbf{r}}, {\mathbf{c}}, {\mathbf{a}}, {\mathbf{d}}} \right)p\left( \mu \right)p\left( b \right)p\left( {\mathbf{t}} \right)p\left( {\left. {\mathbf{r}} \right|\sigma_{r}^{2} } \right)p\left( {\left. {\mathbf{c}} \right|\sigma_{c}^{2} } \right)p\left( {\left. {\mathbf{a}} \right|\sigma_{a}^{2} } \right)p\left( {\left. {\mathbf{d}} \right|\sigma_{d}^{2} ,\lambda } \right)p\left( {\left. {\mathbf{r}} \right|\sigma_{r}^{2} } \right) \\ \end{aligned}$$

$$p\left( {\sigma_{r}^{2} } \right)p\left( {\sigma_{c}^{2} } \right)p\left( {\sigma_{a}^{2} } \right)p\left( {\sigma_{d}^{2} } \right)p\left( \lambda \right)p\left( {\sigma_{e}^{2} } \right),$$

where ${\mathbf{y}}$ is the vector of phenotypic data, $\mu$ is the general mean, $b$ is a covariate with the average SNP homozygosity, ${\mathbf{t}}$, ${\mathbf{r}}$, ${\mathbf{c}}$, ${\mathbf{a}}$ and ${\mathbf{d}}$ are the vectors of order of parity, farm-year-month of farrowing, permanent environmental, additive and dominance effects, respectively. $\sigma_{r}^{2}$, $\sigma_{c}^{2}$, $\sigma_{a}^{2}$, $\sigma_{d}^{2}$ and $\sigma_{e}^{2}$ are the farm-year-month of farrowing, permanent environmental, additive, dominance and residual variance and $\lambda$ is the parameter that reflects the asymmetry of the dominance effects.

The conditional distribution of the data given all the unknowns in the model was:

$$p\left( {\left. {\mathbf{y}} \right|\upmu, b, {\mathbf{t}}, {\mathbf{r}}, {\mathbf{c}},\varvec{ }{\mathbf{a}}, {\mathbf{d}}} \right) = \mathop \prod \limits_{i = 1}^{N} \frac{1}{{\sqrt {2\pi \sigma_{e}^{2} } }}exp\left\{ { - \frac{{\left( {y_{i} - 1\upmu - h_{i} b - {\mathbf{x}}_{{\mathbf{i}}} {\mathbf{t}} - {\mathbf{w}}_{{\mathbf{i}}} {\mathbf{r}} - {\mathbf{q}}_{{\mathbf{i}}} {\mathbf{c}} - {\mathbf{z}}_{{\mathbf{i}}} {\mathbf{a}} - {\mathbf{k}}_{{\mathbf{i}}} {\mathbf{d}}} \right)}}{{2\sigma_{e}^{2} }}} \right\},$$

where $N$ is the number of data, $h_{i}$ is the $i$-*th* element of the vector ${\mathbf{h}}$ of the average SNP homozygosity, and ${\mathbf{x}}_{i}$, ${\mathbf{w}}_{i}$, ${\mathbf{q}}_{i}$, ${\mathbf{z}}_{i}$ and ${\mathbf{k}}_{i}$ are the $i$-*th* rows of the ${\mathbf{X}}$, ${\mathbf{W}}$, ${\mathbf{Q}}$, ${\mathbf{Z}}$ and ${\mathbf{K}}$ matrices that link the phenotypic records with ${\mathbf{t}}$, ${\mathbf{r}}$, ${\mathbf{c}}$, ${\mathbf{a}}$ and ${\mathbf{d}}$, respectively.

The prior distributions for the general mean ($$\upmu$$), the covariate with the average homozygosity ($$b$$), each level of the order of parity effects ($$t_{i} )$$ and the asymmetry parameter ($$\lambda$$) were uniform within appropriate bounds $$\left[ { - {\text{M}},{\text{M}}} \right]$$, $${\text{M}}$$ being a large real number. The prior distributions for $${\mathbf{r}}$$, $${\mathbf{c}}$$, and $${\mathbf{a}}$$ were the products of the following Gaussian distributions:

$$p\left( {\left. {\mathbf{r}} \right|\sigma_{r}^{2} } \right) = \mathop \prod \limits_{i = 1}^{{N_{r} }} \frac{1}{{\sqrt {2\pi \sigma_{r}^{2} } }}exp\left\{ { - \frac{{r_{i}^{2} }}{{2\sigma_{r}^{2} }}} \right\},$$

$$p\left( {\left. {\mathbf{c}} \right|\sigma_{c}^{2} } \right) = \mathop \prod \limits_{i = 1}^{{N_{c} }} \frac{1}{{\sqrt {2\pi \sigma_{c}^{2} } }}exp\left\{ { - \frac{{c_{i}^{2} }}{{2\sigma_{c}^{2} }}} \right\},$$

$$p\left( {\left. \varvec{a} \right|\sigma_{a}^{2} } \right) = \mathop \prod \limits_{i = 1}^{{N_{SNP} }} \frac{1}{{\sqrt {2\pi \sigma_{a}^{2} } }}exp\left\{ { - \frac{{a_{i}^{2} }}{{2\sigma_{a}^{2} }}} \right\},$$

where $N_{r}$, $N_{c}$ and $N_{SNP}$ are the numbers of elements of ${\mathbf{r}}$, ${\mathbf{c}}$, and ${\mathbf{a}}$, respectively. Furthermore, the prior distribution for ${\mathbf{d}}$ was the product of the following skew Gaussian distributions [16]:

$$p\left( {\left. {\mathbf{d}} \right|\sigma_{d}^{2} ,\lambda } \right) = \mathop \prod \limits_{i = 1}^{{N_{SNP} }} \frac{2}{{\sqrt {\sigma_{d}^{2} + \lambda^{2} } }}\phi \left( {\frac{{d_{i} }}{{\sqrt {\sigma_{d}^{2} + \lambda^{2} } }}} \right)\Phi \left( {\frac{\lambda }{{\sigma_{d} }},\frac{{d_{i} }}{{\sqrt {\sigma_{d}^{2} + \lambda^{2} } }}} \right),$$

where $\phi$ and $\Phi$ denote the density function and cumulative distribution function of the standard Gaussian distribution. Following Sahu et al. [16], the first three moments of a skewed Gaussian (${\text{SN}}$) distribution—$x\sim{\text{SN}}\left( {0,\sigma^{2} ,\lambda } \right)$—are:

$$E\left( x \right) = \frac{\sqrt 2 \lambda }{\sqrt \pi },$$

$$E\left[ {x - E\left( x \right)} \right]^{2} = Var\left( x \right) = \sigma^{2} + \lambda^{2} \left( {1 - \frac{2}{\pi }} \right),$$

$$E\left[ {x - E\left( x \right)} \right]^{3} = \lambda^{3} \sqrt {\frac{2}{\pi }} \left( {\frac{4}{\pi } - 1} \right).$$

Note that when *λ* $= 0$, $E\left( x \right) = 0$ and $Var\left( x \right) = \sigma^{2}$, as in the standard Gaussian distribution. Finally, the prior distributions for the variance components $\sigma_{r}^{2}$, $\sigma_{c}^{2}$, $\sigma_{a}^{2}$, $\sigma_{d}^{2}$ and $\sigma_{e}^{2}$ were scaled inverse Chi square distributions:

$$p\left( {\sigma_{x}^{2} |s^{2} ,v} \right) = \chi^{ - 2} \left[ {s^{2} ,v} \right],$$

with parameters $s^{2} = 0$ and $v = - 2$, that correspond to uniform distributions over the positive real scale.

### The Gibbs sampler

The Gibbs sampler is an updating sampling scheme [18] that needs to sample from the full conditional distributions of all unknowns in the model. The full conditional distribution of $\upmu$, $b$ and each level of ${\mathbf{t}}$, ${\mathbf{r}}$, ${\mathbf{c}}$, and ${\mathbf{a}}$ were univariate Gaussian as in the standard implementation of the Gibbs sampler in the mixed model [46]. The conditional distribution of each level of ${\mathbf{d}}$ is the product of a Gaussian distribution with a skewed Gaussian (${\text{SN}}$) distribution with parameters $\lambda$ and $\sigma_{d}^{2}$:

$$\begin{aligned} & p\left( {d_{i} |b,{\mathbf{t}},{\mathbf{r}},{\mathbf{c}},{\mathbf{a}},{\mathbf{d}}_{ - i} ,\sigma_{e}^{2} ,\sigma_{d}^{2} ,\lambda } \right) \\ & = N\left( {\frac{{{\mathbf{k}}_{i} \left( {{\mathbf{y}} - {\mathbf{h}}b - {\mathbf{Xt}} - {\mathbf{Wr}} - {\mathbf{Qc}} - {\mathbf{Za}} - {\mathbf{Kd}}_{ - i} } \right)}}{{{\mathbf{k}}_{i} {\mathbf{k}}_{i} }},\frac{{\sigma_{e}^{2} }}{{{\mathbf{k}}_{i} {\mathbf{k}}_{i} }}} \right){\text{SN}}\left( {0,\lambda ,\sigma_{d}^{2} } \right), \\ \end{aligned}$$

where ${\mathbf{d}}_{ - i}$ is the vector of the dominance effects without the $i$-*th* effect ($d_{i}$), ${\mathbf{k}}_{i}$ is the $i$-*th* row of matrix ${\mathbf{K}}$. In order to facilitate the conditional sampling of $d_{i}$, we invoked the following property of the skewed Gaussian distribution [16]:

$$\begin{aligned} p\left( {x |0,\sigma^{2} ,\lambda } \right) & = \mathop \int \limits_{0}^{\infty } p\left( {x |\sigma^{2} ,\lambda ,u} \right)p\left( u \right)du \\ & = \mathop \int \limits_{0}^{\infty } \frac{1}{{\sqrt {2\pi \sigma^{2} } }}exp\left\{ { - \frac{{\left( {x - \lambda u} \right)^{2} }}{{2\sigma^{2} }}} \right\}\frac{1}{{\sqrt {2\pi } }}exp\left\{ { - \frac{{u^{2} }}{2}} \right\}dt, \\ \end{aligned}$$

which allows to understand the skewed Gaussian distribution as a mixture of an infinite number of standard Gaussian distributions with $\sigma^{2}$ variance and mean defined by $\lambda u$, $u$ being a variable distributed by a half normal (${\text{HN}}$) distribution. This property allows us to define the conditional densities for each level of ${\mathbf{d}}$ as the product of two Gaussian densities:

$$\begin{aligned} & p\left( {d_{i} |b,{\mathbf{t}},{\mathbf{r}},{\mathbf{c}},{\mathbf{a}},{\mathbf{d}}_{ - i} ,\sigma_{e}^{2} ,\sigma_{d}^{2} ,\lambda ,{\mathbf{u}}} \right) \\ & = {\text{N}}\left( {\frac{{{\mathbf{k}}_{i} \left( {{\mathbf{y}} - {\mathbf{h}}b - {\mathbf{Xt}} - {\mathbf{Wr}} - {\mathbf{Qc}} - {\mathbf{Za}} - {\mathbf{Kd}}_{ - i} } \right)}}{{{\mathbf{k}}_{i} {\mathbf{k}}_{i} }},\frac{{\sigma_{e}^{2} }}{{{\mathbf{k}}_{i} {\mathbf{k}}_{i} }}} \right){\text{N}}\left( {\lambda u_{i} ,\sigma_{d}^{2} } \right), \\ \end{aligned}$$

where ${\mathbf{d}}_{ - i}$ is the vector of the dominance effects without the $i$-*th* effect ($d_{i}$), ${\mathbf{k}}_{i}$ is the $i$-*th* row of the ${\mathbf{K}}$ matrix and ${\mathbf{u}} = \left\{ {u_{i} } \right\}$ is the vector of auxiliary variables with the following half normal (${\text{HN}}$) prior distribution:

$$p\left( {\mathbf{u}} \right) = {\text{HN}}\left( {{\mathbf{0}},{\mathbf{I}}} \right).$$

Consequently, the conditional distribution for each element of ${\mathbf{u}}$ was:

$$p\left( {u_{i} |d_{i} ,\lambda } \right) = {\text{HN}}\left( {\frac{{d_{i} \lambda }}{{\lambda^{2} + 1}},\frac{1}{{\lambda^{2} + 1}}} \right),$$

and the conditional distributions for $\lambda$ was:

$$p\left( {\lambda |{\mathbf{d}},{\mathbf{u}},\sigma_{d}^{2} } \right) = N\left( {\frac{{\mathop \sum \nolimits_{i = 1}^{{N_{SNP} }} d_{i} u_{i} }}{{\mathop \sum \nolimits_{i = 1}^{{N_{SNP} }} u_{i}^{2} }},\frac{{\sigma_{d}^{2} }}{{\mathop \sum \nolimits_{i = 1}^{{N_{SNP} }} u_{i}^{2} }}} \right).$$

Furthermore, the conditional distribution for $\sigma_{d}^{2}$ was the following scaled inverted Chi square distributions:

$$p\left( {\sigma_{d}^{2} |{\mathbf{d}},{\mathbf{u}},\lambda } \right) = \chi^{ - 2} \left[ {\left( {{\mathbf{d}} - \lambda {\mathbf{u}}} \right)^{'} \left( {{\mathbf{d}} - \lambda {\mathbf{u}}} \right),N_{SNP} - 2} \right].$$

Finally, the full conditional distribution for $\sigma_{r}^{2}$, $\sigma_{c}^{2}$, $\sigma_{a}^{2}$, and $\sigma_{e}^{2}$ were also the following scaled inverted Chi square distributions:

$$p\left( {\sigma_{r}^{2} |{\mathbf{r}}} \right) = \chi^{ - 2} \left[ {{\mathbf{r^{\prime}r}},N_{r} - 2} \right],$$

$$p\left( {\sigma_{c}^{2} |{\mathbf{c}}} \right) = \chi^{ - 2} \left[ {{\mathbf{c^{\prime}c}},N_{c} - 2} \right],$$

$$p\left( {\sigma_{a}^{2} |\varvec{a}} \right) = \chi^{ - 2} \left[ {{\mathbf{a^{\prime}a}},N_{SNP} - 2} \right],$$

$$p\left( {\sigma_{e}^{2} |{\mathbf{e}}} \right) = \chi^{ - 2} \left[ {{\mathbf{e^{\prime}e}},N_{SNP} - 2} \right],$$

with $$\varvec{e} = {\mathbf{y}} - {\mathbf{1}}\mu - {\mathbf{h}}b - {\mathbf{Xt}} - {\mathbf{Qc}} - {\mathbf{Za}} - {\mathbf{Kd}}$$.

The Gibbs sampler also allows to calculate posterior distributions of the any combination of parameters of the model. Thus, we calculated for each iteration of the Gibbs sampler the following approximations for the additive and dominance genetic variances [47] as:

$$V_{A} = \frac{{\mathop \sum \nolimits_{i = 1}^{{N_{IND} }} s_{ai}^{2} }}{{N_{IND} }} - \left( {\frac{{\mathop \sum \nolimits_{i = 1}^{{N_{IND} }} s_{ai} }}{{N_{IND} }}} \right)^{2} ,$$

$$V_{D} = \frac{{\mathop \sum \nolimits_{i = 1}^{{N_{IND} }} s_{di}^{2} }}{{N_{IND} }} - \left( {\frac{{\mathop \sum \nolimits_{i = 1}^{{N_{IND} }} s_{di} }}{{N_{IND} }}} \right)^{2} ,$$

where $N_{IND}$ is the number of individuals in the population and $s_{ai}$ and $s_{di}$ the breeding and the dominance deviation values for the $i$-*th* individual. They were calculated as:

$$s_{ai} = \mathop \sum \limits_{j = 1}^{{N_{SNP} }} m_{ij} \left( {a_{j} + \left( {1 - 2p_{j} } \right)\left( {\mu_{d} + d_{j} } \right)} \right),$$

$$s_{di} = \mathop \sum \limits_{j = 1}^{{N_{SNP} }} n_{ij} \left( {\mu_{d} + d_{j} } \right),$$

where $\mu_{d} = - \frac{b}{{N_{SNP} }}$ as defined by Xiang et al. [13], $p_{j}$ was the observed allelic frequency for the $j$-*th* SNP and

$$m_{ij} = \left\{ {\begin{array}{*{20}c} {0 - 2p_{j} } &\quad {{\rm if}\;A_{1} A_{1} } \\ {1 - 2p_{j} } &\quad {{\rm if}\;A_{1} A_{2} } \\ {2 - 2p_{j} } &\quad {{\rm if}\;A_{2} A_{2} } \\ \end{array} } \right.,$$

$$n_{ij} = \left\{ {\begin{array}{*{20}c} { - 2\left( {1 - p_{j} } \right)^{2} } &\quad {{\rm if}\;A_{1} A_{1} } \\ {2p_{j} \left( {1 - p_{j} } \right)} &\quad {{\rm if}\;A_{1} A_{2} } \\ { - 2p_{j}^{2} } &\quad {{\rm if}\;A_{2} A_{2} } \\ \end{array} } \right..$$

Note that this parameterization implies a population under Hardy–Weinberg equilibrium and that the covariance between the breeding values and dominance deviations is equal to zero.

Finally, we also calculated the expected inbreeding depression ($I_{D}$) for each percentage of inbreeding as [9, 37]:

$$I_{D} = - \mathop \sum \limits_{j = 1}^{{N_{SNP} }} 2p_{j} \left( {1 - p_{j} } \right)\left( {\mu_{d} + d_{j} } \right)0.01,$$

The computational costs of a Gibbs sampler implementation of the models with 75,000 iterations after a burn-in of the first 25,000 were approximately 100 and 80 CPU hours for populations 1 and 2, respectively. The analysis were performed with single thread process of an Intel(R) Xeon(R) CPU E5-2680 v2 @ 2.80 GHz processor.

### Model comparison

#### Deviance information criterion

The deviance information criterion (DIC) was defined by Spiegelhalter et al. [21]. It compares the global quality of two or more models accounting for model complexity. For a particular model M, the DIC is defined as:

$${\text{DIC}}_{M} = 2\bar{D}_{M} - D\left( {\bar{\theta }_{M} } \right),$$

where $\bar{D}_{M}$ is the posterior expectation of the deviance $D\left( {\theta_{M} } \right)$, and $D\left( {\bar{\theta }_{M} } \right) = - 2{ \log }\left( {p\left( {\mathbf{y}}{|\bar{\theta }_{M} } \right)} \right)$ is the deviance evaluated at the posterior mean estimate of the parameter vector $\left( {\theta_{M} } \right)$. The computation of DIC is composed by two terms, i.e., $\bar{D}_{M}$ is a measure of model fit and $\bar{D}_{M} - D\left( {\bar{\theta }_{M} } \right)$ is related to the effective number of parameters. Models with smaller DIC exhibit a better global fit after accounting for model complexity.

#### Log-marginal probability (logCPO)

If we consider the data vector $= \left( {y_{i} ,{\mathbf{y}}_{ - i} } \right)$, where $y_{i}$ is the $i$-*th* datum and ${\mathbf{y}}_{ - i}$ is the vector of data with $i$-*th* datum deleted, the conditional predictive distribution has a probability density equal to:

$$p\left( {y_{i} |{\mathbf{y}}_{ - i} } \right) = \int p\left( {y_{i} ,\theta |{\mathbf{y}}_{ - i} } \right)p\left( {\theta |{\mathbf{y}}_{ - i} } \right)d\theta ,$$

where $\theta$ is the vector of parameters ($\mu ,b,{\mathbf{t}},{\mathbf{r}},{\mathbf{c}},{\mathbf{a}},{\mathbf{d}},\sigma_{r}^{2}$,$\sigma_{c}^{2} ,\sigma_{a}^{2} ,\sigma_{d}^{2} ,\lambda$ and $\sigma_{e}^{2}$) in the Full Model). Therefore, $p\left( {y_{i} |{\mathbf{y}}_{ - i} } \right)$ can be interpreted as the probability of each datum given the rest of the data, and it is known as the conditional predictive ordinate (CPO) for the $i$-*th* datum. The pseudo log-marginal probability of the data is then:

$$\mathop \sum \limits_{i} ln\,p\left( {y_{i} |{\mathbf{y}}_{ - i} } \right),$$

A Monte Carlo approximation of the CPO suggested by Gelfand [22] is $\mathop \sum \nolimits_{i} ln\,\hat{p}\left( {y_{i} |{\mathbf{y}}_{ - i} } \right)0$, where $\hat{p}\left( {y_{i} |{\mathbf{y}}_{ - i} } \right) = N\left[ {\mathop \sum \nolimits_{j = 1}^{N} \frac{1}{{p\left( {y_{i} |\theta^{j} } \right)}}} \right]^{ - 1}$, and $N$ is the number of Markov chain Monte Carlo (MCMC) draws, and $\theta^{j}$ is the $j$-*th* draw from the posterior distribution of the corresponding parameter. The higher the value of the LogCPO, best fit of the model to data.
