# Supplementary material for: Genomic selection models for directional dominance: an example for litter size in pigs
Source: Genet Sel Evol. 2018 Jan 26;50:1. doi: 10.1186/s12711-018-0374-1 (PMC5787328; doi:10.1186/s12711-018-0374-1)
Supplement: Supplementary file 1 — Additional file 1: Tables S1 and S2. Convergence and effective sample size of the Gibbs sampler. These tables include the results of the required burn-in and required sample size and the estimates of the effective sample size of the Gibbs sampler for Models SN, SC, AN and Full in lines 1 (Table S1) and 2 (Table S2). [file 12711_2018_374_MOESM1_ESM.docx]

**Supplementary Table 1.** Mean (and standard deviation) of the burn-in and required length (RL) to achieve an accuracy of 0.1 for the 0.5 quantile with a 95% of probability and effective sample size (EFS) for heritability (*h^2^*), percentage of dominance variation (*d^2^*), the asymmetry parameter (*λ*) and the covariate with individual heterozygosity (*b*) in Population 1.

|  | | MODEL | | | |
| --- | --- | --- | --- | --- | --- |
|  |  | SN | SC | AN | Full |
| h^2^ | Burn-in | 759.4  (133.8) | 331.4  ( 110.2) | 540.0  (128.9) | 526.4  (126.8) |
|  | RL | 24178.4  (4367.9) | 11168.8 (4010.6) | 17746.0  (4061.5) | 18724.8  (3131.8) |
|  | EFS | 1772.78 | 7488.63 | 2844.53 | 17386.65 |
| d^2^ | Burn-in | 2793.0  (451.4) | 930.0  (83.0) | 5772 .2  (4284.1) | 7628.4  (2781.2) |
|  | RL | 87755.6  (14719.7) | 29392.8 (1675.0) | 181746.6 (133608.8) | 238897.6  (88772.37) |
|  | EFS | 177.09 | 1276.33 | 403.94 | 452.73 |
| *λ* | Burn-in | - | 517.8  (57.93) | - | 8720.2  (1099.96) |
|  | RL | - | 17342.0 (2008.2) | - | 273998.6  (33582.6) |
|  | EFS | - | 1374.03 | - | 84.20 |
| *b* | Burn-in | - | - | 93.0  (53.0) | 9204.0  (1718.8) |
|  | RL | - | - | 3209.8  (1529.0) | 290279.6 (52934.3) |
|  | EFS | - | - | 16510.43 | 140.50 |

**Supplementary Table 2.** Mean (and standard deviation) of the burn-in and required length (RL) to achieve an accuracy of 0.1 for the 0.5 quantile with a 95% of probability and effective sample size (EFS) for heritability (*h^2^*), percentage of dominance variation (*d^2^*), the asymmetry parameter (*λ*) and the covariate with individual heterozygosity (*b*) in Population 2.

|  | | MODEL | | | |
| --- | --- | --- | --- | --- | --- |
|  |  | SN | SC | AN | Full |
| h^2^ | Burn-in | 585.2  (71.5) | 570.4  (126.7) | 601.2  (99.17) | 799.6  (127.6) |
|  | RL | 19064.6  (2058.5) | 18534.4  (3761.2) | 19067.4 (3109.2) | 25685.4  (3911.8) |
|  | EFS | 1355.45 | 2826.81 | 1488.22 | 7035.39 |
| d^2^ | Burn-in | 4461.0  (1255.7) | 1786.2  (366.6) | 5583.8 (1972.4) | 6960.4  (1774.8) |
|  | RL | 140277.4 (39010.7) | 57256.8  (12491.1) | 174991.4 (60660.18) | 218471.4  (58466.4) |
|  | EFS | 81.89 | 337.40 | 108.50 | 255.42 |
| *λ* | Burn-in | - | 890.0  (162.1) | - | 7039.4  (2632.1) |
|  | RL | - | 26880.8  (4908.6) | - | 221202.9  (82693.8) |
|  | EFS | - | 701.38 | - | 185.48 |
| *b* | Burn-in | - | - | 111.8  (23.5) | 5251.8  (3369.39) |
|  | RL | - | - | 4183.2  (876.2) | 162947.6  (103573.3) |
|  | EFS | - | - | 10013.61 | 444.91 |
