# Supplementary material for: Genomic selection models for directional dominance: an example for litter size in pigs
Source: Genet Sel Evol. 2018 Jan 26;50:1. doi: 10.1186/s12711-018-0374-1 (PMC5787328; doi:10.1186/s12711-018-0374-1)
Supplement: Supplementary file 2 — Additional file 2: Tables S3 and S4. Correlations between estimates from Models SN, AC, AN and Full. These tables present the correlations between estimates of SNP additive (a) and dominance (b) effects, individual breeding values (c) and dominance deviations (d) with Models SN, SC, AN and Full in lines 1 (Table S3) and 2 (Table S4). [file 12711_2018_374_MOESM2_ESM.docx]

**Supplementary Table 3.** Correlation between estimates of SNP additive (a) and dominance (b) effects, individual breeding values (c) and dominance deviations (d) with the SN, SC, AN and Full Models in population 1.

1. SNP additive effects

|  | SN | SC | AN | Full |
| --- | --- | --- | --- | --- |
| SN | - | 0.992 | 0.993 | 0.995 |
| SC | - | - | 0.991 | 0.998 |
| AN |  |  | - | 0.997 |

1. SNP dominance effects

|  | SN | SC | AN | Full |
| --- | --- | --- | --- | --- |
| SN | - | 0.989 | 0.990 | 0.991 |
| SC | - | - | 0.988 | 0.999 |
| AN |  |  | - | 0.994 |

1. Breeding values

|  | SN | SC | AN | Full |
| --- | --- | --- | --- | --- |
| SN | - | 0.933 | 0.944 | 0.938 |
| SC |  | - | 0.998 | 0.999 |
| AN |  |  | - | 0.999 |

1. Dominance deviations

|  | SN | SC | AN | Full |
| --- | --- | --- | --- | --- |
| SN | - | 0.987 | 0.769 | 0.884 |
| SC |  | - | 0.856 | 0.944 |
| AN |  |  | - | 0.978 |

**Supplementary Table 4.** Correlation between estimates of SNP additive (a) and dominance (b) effects, individual breeding values (c) and dominance deviations (d) with the SN, SC, AN and FULL models in population 2.

1. SNP additive effects

|  | SN | SC | AN | FULL |
| --- | --- | --- | --- | --- |
| SN | - | 0.996 | 0.997 | 0.994 |
| SC | - | - | 0.996 | 0.999 |
| AN | - | - | - | 0.993 |

1. SNP dominance effects

|  | SN | SC | AN | FULL |
| --- | --- | --- | --- | --- |
| SN | - | 0.994 | 0.997 | 0.990 |
| SC | - | - | 0.994 | 0.999 |
| AN | - | - | - | 0.990 |

1. Breeding values

|  | SN | SC | AN | FULL |
| --- | --- | --- | --- | --- |
| SN | - | 0.794 | 0.842 | 0.777 |
| SC | - | - | 0.994 | 0.999 |
| AN | - | - | - | 0.988 |

1. Dominance deviations

|  | SN | SC | AN | FULL |
| --- | --- | --- | --- | --- |
| SN | - | 0.951 | 0.702 | 0.970 |
| SC | - | - | 0.857 | 0.773 |
| AN | - | - | - | 0.989 |
