# Supplementary material for: Genomic selection models for directional dominance: an example for litter size in pigs
Source: Genet Sel Evol. 2018 Jan 26;50:1. doi: 10.1186/s12711-018-0374-1 (PMC5787328; doi:10.1186/s12711-018-0374-1)

**Supplementary Figure 1.** Plots of the five Gibbs sampler chains (1 –gray- , 2 –red-, 3 –green-, 4 –blue- and 5 -cyan-) for the covariate with individual heterozygosity (*b*) and the asymmetry parameter (*λ*) and bivariate plot of the Gibbs samples for the Full model in Population 1.


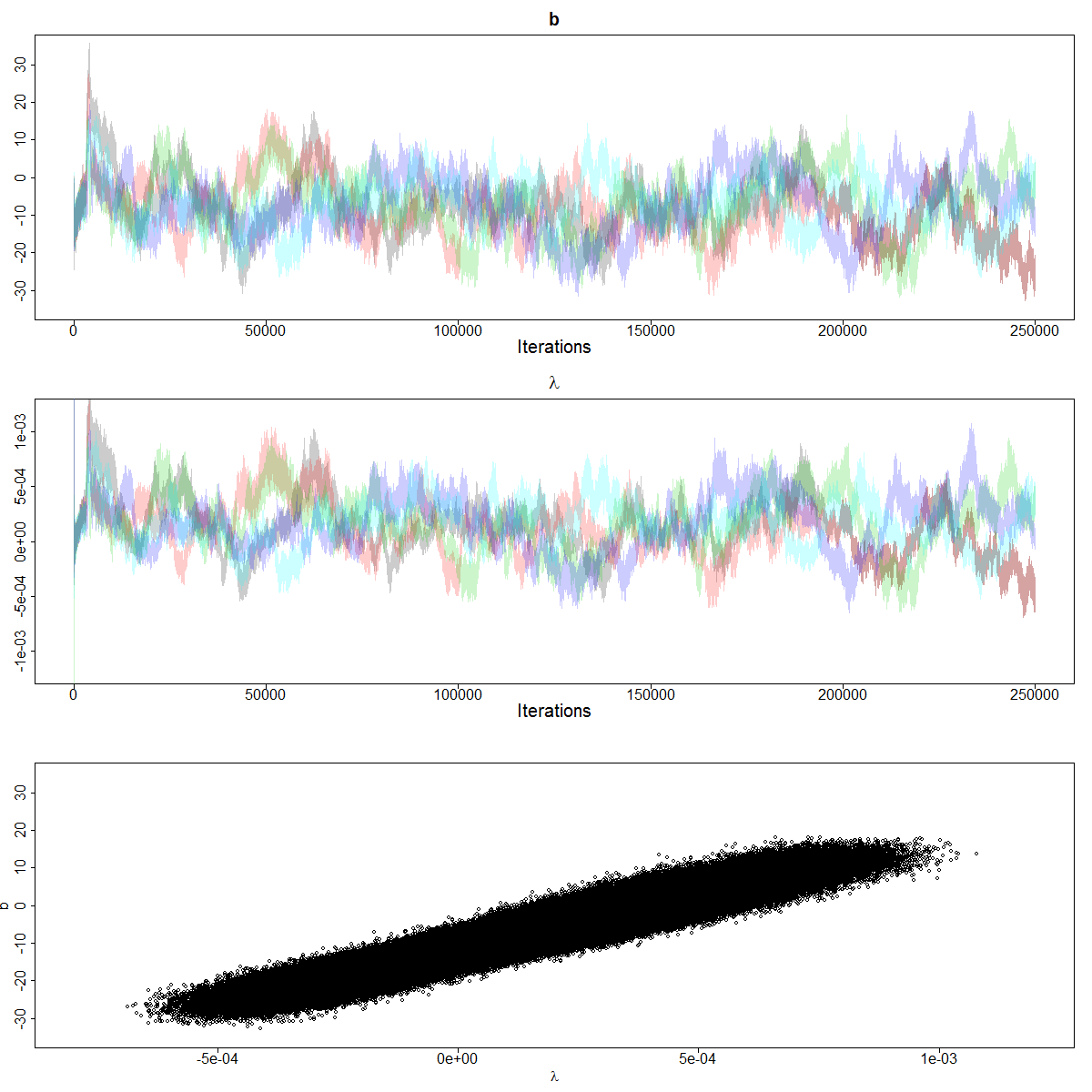


**Supplementary Figure 2.** Plots of the five Gibbs sampler chains (1 –gray- , 2 –red-, 3 –green-, 4 –blue- and 5 -cyan-) for the covariate with individual heterozygosity (*b*) and the asymmetry parameter (*λ*) and bivariate plot of the Gibbs samples for the Full model in Population 2.


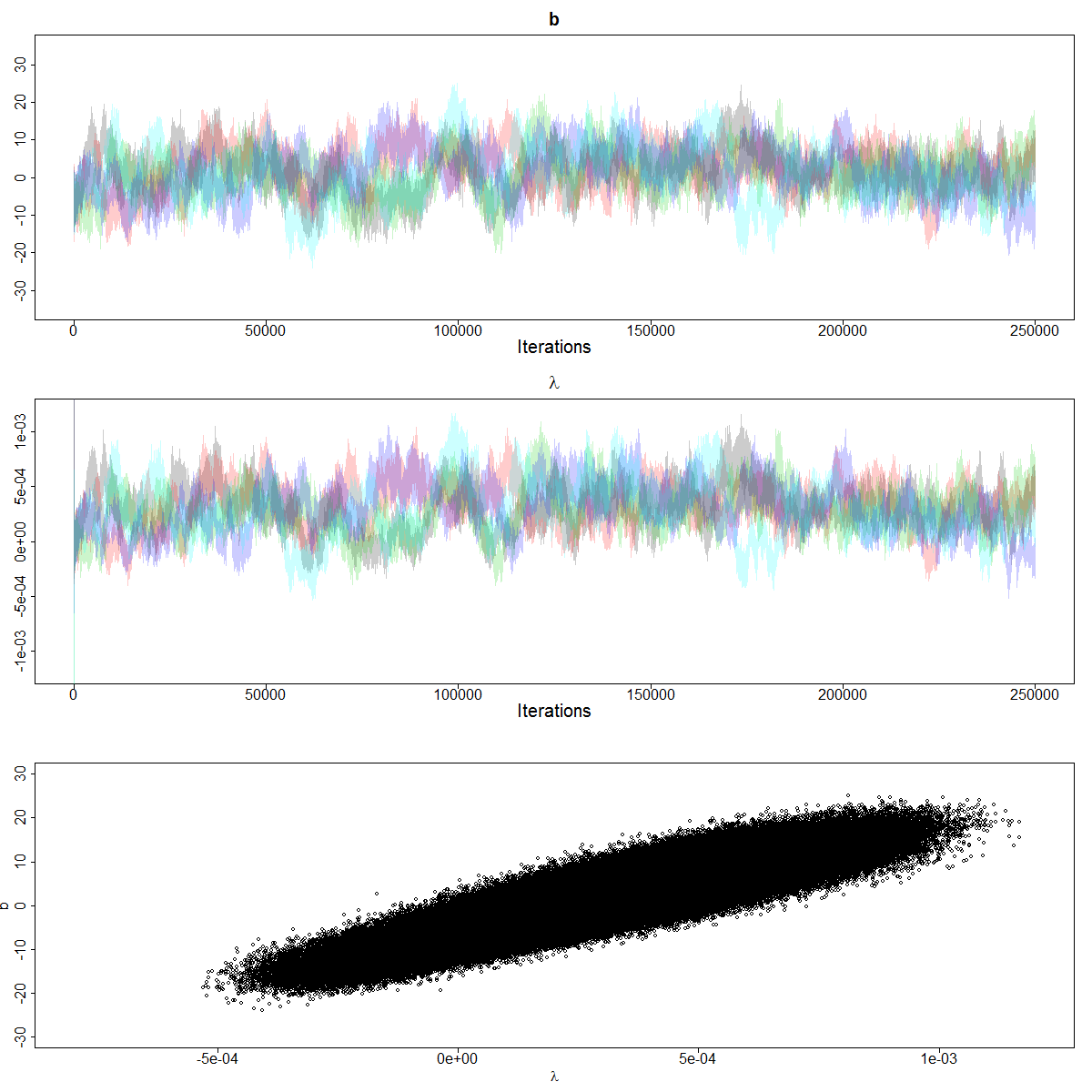

Supplement: Supplementary file 3 — Additional file 3: Figures S1 and S2. Plots of the Gibbs sampler chains for the Full Model. These figures include the plots of the five Gibbs sampler chains for the covariate with individual heterozygosity (b) and the asymmetry parameter (λ) and bivariate plots of the Gibbs samples for the Full Model in lines 1 (Figure S1) and 2 (Figure S2). [file 12711_2018_374_MOESM3_ESM.docx]
